# Factitious Disorders and Cardiothoracic Surgery: The Ongoing Multidisciplinary Challenges of a Complex Case

**DOI:** 10.1155/2009/103265

**Published:** 2009-12-08

**Authors:** Michael S. Firstenberg, John H. Sirak, Benjamin Sun, David P. Kasick

**Affiliations:** ^1^Division of Cardiothoracic Surgery, The Ohio State University Medical Center, Columbus, OH 43210, USA; ^2^Department of Psychiatry, The Ohio State University Medical Center, Columbus, OH 43210, USA

## Abstract

Chronic factitious disorder, Munchausen's syndrome, can be challenging to manage—particularly when complaints and symptoms suggest medical or surgical emergencies. We present a patient whose problems have spanned many years and a great distance. Hopefully, with a greater awareness of this disease, as this patient continues to seek health care in many different hospitals, the implications of timely access to information, good histories and physical exams, and an index of suspicion can assist in potentially avoiding unnecessary, expensive, and invasive evaluations.

## 1. Introduction

Patients with factitious disorder can be challenging to manage, particularly when their complaints prompt concerns for surgical emergencies and they repeatedly present to different medical centers for treatment of the same problem. We report our ongoing experiences with a patient, who has been described by others at an institution over 1000 miles away. In review, clearly his sophistication, education, and pathology have progressed over time. Hopefully, with a greater awareness of pathology, appropriate care can be provided while avoiding unnecessary, expensive, and invasive evaluations. 

Chambers and colleagues described a 20-year-old man who presented prior to 2005 to an institution in New Mexico with severe ripping chest pain radiating to his back [1]. He gave a history of Ehler-Danlos syndrome and a medically managed Type I aortic dissection. His family history included multiple deaths related to aortic aneurysms and provided prescriptions for antihypertensives and reported multiple allergies. Verbal reports described recent MRI and echo findings suspicious for an intimal flap in the ascending aorta—however images were not available at the time. Based upon his presentation, he underwent surgical exploration. The intraoperative findings showed no evidence of an aortic dissection. Aortic wall biopsy was unremarkable for acute or chronic pathology. His uncomplicated postoperative course was challenged by multiple complains and exhaustive testing—all reported to be negative (including a brain MRI). Past medical records indicated a pattern of hospital admissions across multiple states with similar complaints and emergent evaluations. Ultimately, after finding no physical abnormalities, he was transferred to a psychiatric facility and was subsequently discharged.

Recently (03/2009), we believe the same individual, a 28-year-old African-American, thin, male with a reported history of a heart transplant, Ehler-Danlos syndrome, and a medically managed Type A aortic dissection presented to an outside hospital with complaints of severe ripping chest pain radiating to his back. Emergent transfer to our center was arranged. Upon arrival, he was normotensive with a heart rate in the 80's. His physical exam, including a detailed neurological, vascular, and musculo-skeletal exam, was unremarkable, except for a well-healed sternal incision. A chest x-ray showed median sternotomy wires, but no other radio-opaque markers. His blood work was normal except for a cyclosporine level of 68. He gave an addition history of a Stage 3 Glioblastoma multiforme with multiple cerebral aneurysm coils and said he could not have MRIs. He also reported allergies to multiple medications, contrast, and MRI dye. He claimed his heart transplant had been performed in Germany after spending many months in the ICU following a massive heart attack that required him to be supported on a ventricular assist device. He did not recall the name of the hospital or the surgeon caring for him. He also gave a history of a Type A aortic dissection managed medically as he was told he would not survive surgery. He returned to the United States and reported he was receiving care in multiple Pennsylvania hospitals. Following appropriate release of medical information authorization, calls to the named hospitals confirmed that he had multiple visits, but they had no reports of him having received a heart transplant or any other major problems.

The cardiologist on call at our facility recalled performing a normal transesophageal echo on a similar patient, but with a slightly different name, a month previously. This realization led to further investigations, which suggested that this patient had likely presented to our institution with similar complaints and histories several times over the past six years, having registered under slightly different names and birthdates. Despite blood work evidence that he was taking cyclosporine, we could not identify the prescribing physician nor could we confirm, other than the x-ray findings and sternotomy scar, that he had ever had any cardiac surgery—let alone a heart transplant.

Concern for a chronic factitious disorder (Munchausen's syndrome) prompted a psychiatric consultation. He provided medical records from several hospitals across the Midwest and eastern United States, which he felt was evidence of his conditions. Occasionally, he had rapid, pressured speech, limited impulse control, suspiciousness, irritability, anger, and was often provocative in demanding that various physicians at the hospital should meet with him to address the various medical problems he perceived he had. Frequently making notes about encounters with the staff, at one point he was observed to even have been writing copiously on his bed sheets. Despite his intense preoccupation and goal-directed behavior regarding his health, he frequently limited or refused the interventions necessary to further address his concerns. He refused to allow us to contact any family members after elaborate explanations as to why they would not be available and when he also told us that he was a traveling minister, he also refused to allow us to contact his reported employer.

He did agree to take antipsychotic medications, which improved his symptoms. After an extensive negative work-up, he was subsequently transferred to an inpatient psychiatric facility and was discharged after a short period of observation.

## 2. Discussion

Munchausen's syndrome, originally described in 1951, consists of recurrent hospitalization, peregrination (travel), and pseudologica fantastica [2]. In discussion with the authors of the original report [1], we are convinced that he is the same individual. We believe that our patient's behavior, with his history of extensive evaluations and invasive procedures, including emergent thoracic surgery, certainly qualifies for this severe, chronic subtype of factitious disorder.

Our patient also exemplifies the inherent challenges of understanding patients with factitious disorder. While the externally observable behaviors of these patients may contain similarities, the internal gains, personality structure, and psychological needs of these patients are diverse. Additionally, the presence of factitious symptoms does not exclude the presence of other severe forms of comorbid mental illness [3], and does not exclude the possibility that a proportion of these patients may actually suffer from somatic delusions.

This case also illustrates the importance of a thorough history and physical. Clinically he had no physical findings that correlated with his medical history (except for the sternotomy incision). While heart transplants have been performed in patients with connective tissue disorders, such as Marfan's [4], the likelihood that a U.S. citizen with these problems would have received a heart transplant in a foreign country would be highly unusual. Nevertheless, even young patients—and those with multiple medical problems, can have unusual and even catastrophic problems [5]. But even a little misleading information can lead the most astute of clinicians down the wrong pathway when faced with a potentially emergent life-threatening problem.

While the case of a different individual patient with Munchausen's disorder has been reported by multiple authors in New York City in the early 1990's [6–8], to our knowledge, this is the first describing the same patient over such a geographic distance and period of time. While there appears to be a progression of his pathology in terms of his history, other aspects of his presentation and behavior remain remarkably consistent. We hope that describing this case may assist those who may encounter him, or similar patients, in the future to better understand this problem and minimize the risk of further iatrogenic harm.
